# Acknowledgement to Reviewers of *Antioxidants* in 2013

**DOI:** 10.3390/antiox3010114

**Published:** 2014-02-28

**Authors:** Antioxidants Editorial Office

**Affiliations:** MDPI AG, Klybeckstrasse 64, CH-4057 Basel, Switzerland

The editors of *Antioxidants* would like to express their sincere gratitude to the following reviewers for assessing manuscripts in 2013: 

Abbas, S.De Tullio, M.C.Loizzo, Monica R.Abdel-Aal, El-sayed M.Dean, Lisa Ludewig, GabrieleAlves, AndreiaDurazzo, AlessandraMalathy, P.Andini, RitaEspley, Richard V.Malta, Luciana GomesAndreotti, CarloEstevinho, LeticiaMedina, Miguel A´ngelApak, ReşatFailla, MarkMiceli, AntonioAruoma, OkezieFarid, ChematMiguel, M. G.Baiano, A.Franco, RodrigoMiller, Kenneth BBarrajón-Catalán, EnriqueFry, JefferyMimica-Dukic, NedaBattino, MaurizioGiannenas, I.Ming, Li-juneBenavente-Garcia, O.Gordon, MichaelMorales, Juan C.Bendini, AlessandraGoya, LuisMosley, Robert LeeBöhm, VolkerHadjipavlou-Litina, DimitraMullen, BillBrambilla, A.Haleagrahara, NagarajaNemzer, BorisBrantner, AdelheidHarris, CoryNiki, EtsuoCalokerinos, AnthonyInnocenti, MarziaÖzyürek, MustafaCarvalho, PauloIriti, MarceloPaliyath, GopinadhanCeli, PietroJastrebova, JelenaPanzella, LuciaCéspedes, Carlos L.Kaiser, AndreaPérez-Jiménez, JaraChen, Ching-YiKhan, M. FirozePizzala, RobertoChoi, Young HaeKhandaker, LailaPolidoros, AlexiosClifford, MikeKremer, DarioPolikarpov, IgorCoelho, José A.Liakopoulou-Kyriakides, MariaPriefer, RonnyDalessandro, GiuseppeProestos, CharalamposDe Bellis, LuigiLiu, WenlanPyrzynska, KrystynaRagaee, S. M.Sarker, SatyaStriker, Gary E.Rahman, AnisurSato, KenjiSureda, AntoniRomain, CindySaul, NadineTundis, RosaRomano, AnabelaSchmeda Hirschmann, GuillermoUdenigwe, Chibuike C.Rouanet, Jean-MaxUlrichová, JitkaRoychowdhury, SanjoySenchina, DavidVadigepalli, RajanikanthRuiz-Sanz, José IgnacioSerrano, MaríaVieira, A.Rupasinghe, H.P. VasanthaSommano, SaranaWeber, John T.Ryan, Michael J.Spanier, BrittaWilliams, Robert J.Saleh, Mahmoud A.Stevanovic, TatjanaZhang, JunzengSalvador, MirianStich, Karl

